# Refining Automatically Extracted Knowledge Bases Using Crowdsourcing

**DOI:** 10.1155/2017/4092135

**Published:** 2017-05-14

**Authors:** Chunhua Li, Pengpeng Zhao, Victor S. Sheng, Xuefeng Xian, Jian Wu, Zhiming Cui

**Affiliations:** ^1^School of Computer Science and Technology, Soochow University, Suzhou 215006, China; ^2^Computer Science Department, University of Central Arkansas, Conway, AR, USA; ^3^College of Computer Engineering, Suzhou Vocational University, Suzhou 215104, China

## Abstract

Machine-constructed knowledge bases often contain noisy and inaccurate facts. There exists significant work in developing automated algorithms for knowledge base refinement. Automated approaches improve the quality of knowledge bases but are far from perfect. In this paper, we leverage crowdsourcing to improve the quality of automatically extracted knowledge bases. As human labelling is costly, an important research challenge is how we can use limited human resources to maximize the quality improvement for a knowledge base. To address this problem, we first introduce a concept of semantic constraints that can be used to detect potential errors and do inference among candidate facts. Then, based on semantic constraints, we propose rank-based and graph-based algorithms for crowdsourced knowledge refining, which judiciously select the most beneficial candidate facts to conduct crowdsourcing and prune unnecessary questions. Our experiments show that our method improves the quality of knowledge bases significantly and outperforms state-of-the-art automatic methods under a reasonable crowdsourcing cost.

## 1. Introduction

There are numerous information extraction projects that use a variety of techniques to extract knowledge from large text corpora and World Wide Web [1]. Example projects include YAGO [2], DBPedia [3], NELL [4], open information extraction [5], and knowledge vault [6]. These projects provide automatically constructed knowledge bases (KBs) with massive collections of entities and facts, where each entity or fact has a confidence score. However, machine-constructed knowledge bases contain noisy and unreliable facts due to the variable quality of information and the limited accuracy of extractors. Transforming these candidate facts into useful knowledge is a formidable challenge [7].

To alleviate the amount of noise in automatically extracted facts, these projects often employ ad hoc heuristics to reason about uncertainty and contradictoriness due to the large scale of the facts. There exists significant work in developing effective algorithms to perform joint probabilistic inference over candidate facts [7, 8]. Automated approaches have been improved in terms of quality but remain far from perfect. Therefore, effective methods to obtain high quality knowledge are desired. It is easy for human experts to determine whether a fact is correct or not. However, it is impossible to hire experts to correct all of them. Recently, due to the availability of Internet platforms like Amazon Mechanical Turk (MTurk), which enables the participation of human workers in a large scale, crowdsourcing has been proven to be a viable and cost-effective alternative solution. Crowdsourcing is normally used to create labelled datasets to apply machine learning algorithms and becomes an effective way to handle computer-hard tasks [9–11], such as sentiment analysis [12], image classification [13], and entity resolution [14]. The limitations of machine-based approaches and the availability of easily accessible crowdsourcing platforms inspire us to exploit crowdsourcing to improve the quality of automatically extracted knowledge bases.

In this paper, we study the problem of refining knowledge bases using crowdsourcing. Specifically, given a collection of noisy extractions (entities and their relationships) and a budget, we can obtain a set of high quality facts from these extractions via crowdsourcing. In particular, there are two subproblems to address in this study: (1) error Detection: how can we effectively detect potential erroneous candidate facts which need to be verified by the crowd? Information extraction systems are able to extract massive collections of interrelated facts. Some facts are correct, while others are clearly incorrect and contradictory. Asking humans to verify all candidate facts is generally not feasible due to the large size of extractions. Hence, one of key challenges is to determine which subset of knowledge should be presented to the crowd for verification. (2) Knowledge Inference: how can we accurately infer consistent knowledge based on crowd feedbacks? Errors introduced from the extraction process cause inconsistencies in the knowledge base, which may contain duplicate entities and violate key ontological constraints such as subsumption, mutual exclusion, inverse, and domain and range constraints.

To address these problems, we first introduce a concept of semantic constraints, which is similar to integrity constraints in data cleaning. Then we propose rank-based and graph-based algorithms to judiciously select candidate facts to conduct crowdsourcing based on semantic constraints. Our method automatically assigns the most “beneficial” task to the crowd and infers the answers of some candidate facts based on crowd feedbacks. Experiments on NELL's knowledge base show that our method can significantly improve the quality of knowledge and outperform state-of-the-art automatic methods under a reasonable crowdsourcing cost.

To summarize, we make the following contributions:

1. We propose a rank-based crowdsourced knowledge refining framework. We introduce a concept of semantic constraints and utilize it to detect potential contradictive facts. We present a score function taking both uncertainty and contradictoriness into consideration to select the most beneficial candidate facts for crowdsourcing.
2. We construct a graph based on the semantic constraints and utilize the graph to ask questions and infer answers. We judiciously select candidate facts to ask in order to minimize the number of candidate facts to conduct crowdsourcing. We propose path-based and topological-sorting-based algorithms that ask multiple questions in parallel in each iteration.
3. We develop a probability-based method to tolerate the errors introduced by the crowd and propagated through inference rules.
4. We conduct experiments using real-world datasets on a real crowdsourcing platform. Experimental results show the effectiveness of the proposed approaches.

The rest of this paper is structured as follows. We first review related work in Section 2 and introduce basic concepts related to our work in Section 3. Then we describe our proposed approaches in Section 4. We report experimental results in Section 5 and conclude in Section 6.

## 2. Related Work

Information extraction techniques are widely applied in the construction of web-scale knowledge bases. In this paper, we use Never Ending Language Learner (NELL) [4] as a case study. NELL starts from a few “seed instances” of each category and relation and generates a knowledge base iteratively. It uses natural language processing and information extraction techniques to extract candidate facts from a large web corpus, using facts learned from the previous iteration as training examples. NELL has four subcomponents that extract candidate facts, namely, Pattern Learner, SEAL, Morphological Classified, and Rule Learner. NELL uses heuristics and ontological constraints to promote candidate facts into a knowledge base, assigning each promotion a confidence value.

Early work on cleaning a noisy knowledge base was considered by Cohen et al. [15]. They considered only a small subset of KB errors. Jiang et al. [8] proposed a method for cleaning knowledge bases at a broader scope using Markov Logic Networks (MLNs). This method performs joint probabilistic inference over candidate facts. To make inference and learning tractable, Jiang et al. surmounted these obstacles with a number of approximations and demonstrated the utility of joint reasoning in comparison to a baseline that considers each fact independently. More recently, Pujara et al. [7] improved the model of Jiang et al. by including multiple extractors and reasoning about coreferent entities. Furthermore, Pujara et al. used probabilistic soft logic (PSL) to avoid scalability limitation of MLNs. Dong et al. [6] employed supervised machine learning methods for fusing distinct information sources by combining noisy extractions from the web with prior knowledge derived from existing knowledge repositories. However, all of above methods are automated algorithms and do not leverage the power of crowdsourcing.

There also exist many research works that incorporate crowdsourcing into data and knowledge management, such as data cleaning [14, 16–19], record linkage [20, 21], schema matching [22–25], and knowledge acquisition [26, 27]. For example, Wang et al. [14] proposed CrowdER to solve the problem of entity resolution via crowdsourcing. Zhang et al. [16] used crowdsourcing to clean uncertain data. Chu et al. [18] proposed KATARA, a data cleaning system that utilizes the power of knowledge bases and crowdsourcing to clean tables. Demartini et al. [20] proposed ZenCrowd which uses a mixed human-machine workflow to solve the entity linking problem. Gokhale et al. [21] studied how to do hands-off crowdsourcing record linkage which requires no involvement of developers. Sarasua et al. [22] studied the problem of ontology matching using crowdsourcing. Fan et al. [23] proposed a hybrid machine-crowdsourcing system for matching web tables. Kondreddi et al. [26, 27] developed HIGGINS, a framework for human intelligence games for knowledge acquisition, to expand and complement the output of automated information extraction methods. However, so far, there has been little discussion about how to use crowdsourcing to clean a noisy knowledge base with semantic constraints.

## 3. Preliminaries

### 3.1. Knowledge Bases

We consider an automatically extracted knowledge base as a probabilistic knowledge base, which stores facts in a form of triple (subject, predicate, and object), for example, (Brussel, citycapitalofcountry, Belgium). Each fact *t*_*i*_ has a confidence score, representing the probability that the corresponding information extraction system “believes” the fact is correct. We formally define an extracted knowledge base as follows.

**Definition 1 .**

An extracted knowledge base (KB) is a 5-tuple *K* = (*ℰ*, *C*, *R*, Π, *L*), where

1. *ℰ* = {*e*_1_,…, *e*_|*ℰ*|_} is a set of entities. Each entity *e* ∈ *ℰ* refers to a real-world object.
2. *C* = {*c*_1_,…, *c*_|*C*|_} is a set of categories (or types). Each category *c* ∈ *C* is a subset of *ℰ*. Each entity *e* ∈ *ℰ* belongs to one or more categories.
3. *R* = {*r*_1_,…, *r*_|*R*|_} is a set of predicates. Each *r* ∈ *R* defines a binary relation between one or more pairs of types. For example, the *wasBornIn* predicate specifies a binary relation between *Person* and *Place*. We call the types of subject and object domain and range of the predicate, respectively.
4. Π = {(*t*_1_, *p*_1_),…, (*t*_|Π|_, *p*_|Π|_)} is a set of weighted facts. For each (*t*, *p*) ∈ Π, *t* is a triple (*x*, *r*, *y*) representing a fact that relation *r* holds between *x* and *y*, where *x*, *y* ∈ *ℰ*, *r* ∈ *R*; *p* ∈ *ℝ* is a weight indicating the probability that an information extraction system believes the corresponding fact is correct, that is, a confidence score.
5. *L* = {*F*_1_,…, *F*_|*L*|_} is a set of ontological relations. It defines concept hierarchy and semantic relationships between categories and relations.

The definition of *C* implies a concept hierarchy: for any *c*_*i*_, *c*_*j*_ ∈ *C*, *c*_*i*_ is subclass of *c*_*j*_ if and only if *c*_*i*_⊆*c*_*j*_. Typing provides semantic context for extracted entities and is commonly adopted by the information extraction systems, so we make it an integral part of the definition. We use *Cat*(*x*, *c*) to denote that *x* is an entity of category *c* and use *Rel*(*x*, *y*, *r*) to denote that relation *r* holds between entities *x* and *y*.

An automatically extracted knowledge base could be very large and noisy. For example, the knowledge vault [6] has 1.6B triples, of which 324M have a confidence of 0.7 or higher, and 271M have a confidence of 0.9 or higher. NELL so far has acquired a knowledge base with over 80M confidence-weighted facts, 2M of which have a confidence of 0.9 or higher. The overall estimated precision of NELL's promoted facts across first 66 iterations is 74%.

### 3.2. Crowdsourcing

There exist a number of crowdsourcing platforms, such as MTurk and CrowdFlower. In such platforms, we can ask human “workers” to complete microtasks. For example, we may ask them to answer questions like “Is Italy a country?” Each microtask is referred as a human intelligent task (HIT). After having completed a HIT, a worker is rewarded with a certain amount of money based on the difficulty of the HIT. That is, invoking the crowd for knowledge cleaning comes with a monetary cost. In addition, a human worker may not always produce a correct answer for a HIT. To mitigate such human errors, we assign each HIT to multiple workers and then take a majority vote. However, even when majority votes are used, we may still get incorrect answers from the crowd. As a consequence, it is crucial to take human errors into account when designing a crowd-based algorithm.

Given a set of candidate facts to be sent to the crowd, we need to combine them into HITs. For each fact, the crowd needs to verify whether the fact is correct or not. We have four questions as one HIT, where each question contains a candidate fact requiring workers to verify its correctness.  Box 1 shows an example of questions we generate as an HIT for MTurk. A brief description of the HIT is shown at the top. To assist workers to understand the fact, we provide a description of the related category or relation and use a human format for each fact.

## 4. Methodology

Our method takes an automatically extracted knowledge base as input and identifies a set of true facts from noisy extractions through crowdsourcing. We first introduce the concept of semantic constraints that can be used to detect potential erroneous facts and do inference among candidate facts. And then we propose a score function to measure the usefulness of candidate facts, in order to conduct crowdsourcing. In Section 4.3, we will explain how to leverage semantic constraints as inference rules to prune unnecessary questions. Finally, we will discuss our error-tolerant techniques.

### 4.1. Semantic Constraints

Integrity constraints are effective tools used in data cleaning. This section introduces a similar concept called semantic constraints that can be used to clean noisy knowledge bases. These constraints can be learned from training data or derived from ontological constraints. The ontological constraints can be seen as axioms or rules in first-order logic. For example, we can represent an ontological constraint (every *Athlete* is a *Perso*n) with a rule, *Athlete*(*x*)⇒*Person*(*x*). Similarly, since a *City* is not a *Person*, we can have a following rule, City(*x*)⇒¬*Person*(*x*).

We derive semantic constraints according to ten types of ontological relations used in NELL: subsumption among categories and relations (e.g., every bird is an animal); mutually exclusive categories and relations (e.g., no person is a location); inversion (for mirrored relations like TeamHasPlayer and PlaysForTeam); the type of the domain and range of each predicate (e.g., the mayor of a city must be a person); the functionality of relations (e.g., a person has only one birth date); antisymmetric (e.g., if person *a* writes book *b*, then *b* cannot write *a*); and antireflexive (e.g., a company cannot produce itself).

We use the following notations: *Sub* and *RSub* for subclass relationships; *Mut* and *RMut* for mutual exclusion relationships; *Dom* and *Ran* for domain and range relationships; Inv for inversion; *Fun* for functionality, *AntiSym* for antisymmetric, and *AntiRef* for antireflexive.

There are two types of semantic constraints according to the label transitive relation between candidate facts:* contradictive relation* and* positive relation*. The derived semantic constraints are shown as follows.

*Contradictive Relation*. In semantic constraints of this type, if a candidate fact is correct, we can infer that another candidate fact must be incorrect. Violations of contradictive relations indicate potential errors.

(1) Mutc1,c2∧Catx,c1⟹¬Catx,c2

(2) RMutr1,r2∧Relx,y,r1⟹¬Relx,y,r2

(3) Mutc1,c2∧Domr,c1∧Catx,c2⟹¬Relx,y,r

(4) Mutc1,c2∧Domr,c1∧Relx,y,r⟹¬Catx,c2

(5) Mutc1,c2∧Ranr,c1∧Catx,c2⟹¬Rely,x,r

(6) Mutc1,c2∧Ranr,c1∧Rely,x,r⟹¬Catx,c2

(7) Funr∧Relx,y,r⟹¬Relx,z,r

(8) AntiRefr⟹¬Relx,x,r

(9) AntiSymr∧Relx,y,r⟹¬Rely,x,r

*Positive Relation*. In semantic constraints of this type, if a candidate fact is correct, we can infer another candidate fact is also correct.

(10) Subc1,c2∧Catx,c1⟹Catx,c2

(11) RSubr1,r2∧Relx,y,r1⟹Relx,y,r2

(12) Invr1,r2∧Relx,y,r1⟹Rely,x,r2

(13) Domr,c∧Relx,y,r⟹Catx,c

(14) Ranr,c∧Relx,y,r⟹Caty,c

Given semantic constraints and a set of candidate facts, we will generate a set of ground rules. A ground rule is a rule containing only candidate facts and no variables. We first instantiate a formula of semantic constraint using ontological relations and candidate facts in the knowledge base. Then, we omit the part of instantiated ontological relations since they are deemed to be true and obtain ground rules containing only candidate facts. For example, (a) in Box 2 are sample ontological relations defined in NELL. Considering candidate facts (b) in Box 2, corresponding ground rules generated according to semantics constraints are shown (c) in Box 2.

While contradictive semantic constraints can be used to detect potential erroneous facts, both positive constraints and contradictive constraints can be used to do inference among candidate facts.

### 4.2. Ranking Facts Based on Benefit

In this section, we propose a rank-based method for knowledge refining. We would like to select the most beneficial candidate facts to conduct crowdsourcing under a given budget for a knowledge base. It is obvious that we prefer to choose the facts that the corresponding information extraction system is most uncertain about. In addition, the facts that violate semantic constraints the most are of high risk and important ones to the knowledge base. It is beneficial to verify them via crowdsourcing. In this paper, we will use the contradictoriness to estimate the risk and importance of candidate facts. In summary, we will first evaluate the benefit of candidate facts in terms of improving the quality of the knowledge base by taking both uncertainty and contradictoriness into consideration. Then we will rank them according to their evaluation scores and choose top *k* facts to conduct crowdsourcing.

*Uncertainty Score.* A threshold *τ* is often applied to probabilistic extractions. Facts with a high confidence can be assumed to be correct, while facts with a confidence less than threshold *τ* are deemed to be most likely incorrect. The most uncertain facts are those whose probability are closest to threshold *τ*. We use *conf*_*m*_(*t*_*i*_) to denote the machine-based probability estimation of a fact *t*_*i*_ being correct. Therefore, we model the uncertainty of a fact *t*_*i*_ as follows.

(15) $$\begin{array}{c} Uncertainty\left(\;t_{i}\;\right)=1-\left|\;conf_{m}\left(\;t_{i}\;\right)-\tau \;\right|. \end{array}$$

The information extraction systems commonly provide a confidence score for each candidate fact, that is, the weight *p* in the knowledge base definition. We adopt the weight *p*_*i*_ as the machine-based probability estimation of a fact *t*_*i*_ being correct.

(16) $$\begin{array}{c} conf_{m}\left(\;t_{i}\;\right)=p_{i}. \end{array}$$

Information extraction systems usually use many different extraction techniques to generate candidates. For example, NELL produces separate extractions from lexical, structural, and morphological patterns. If the patterns used to extract each candidate fact are provided, this extra information can help us better estimate the probability. We can use a simple logistic regression model learned from training data to predict the probability of each candidate fact being correct [28, 29]. The features are whether each pattern cooccurs with the candidate fact, and the coefficients reflect the reliability of patterns.

*Contradictoriness Score.* Based on contradictive semantic constraints introduced in Section 4.1, we can detect inconsistency, errors, and conflicts among candidate facts as the violations of these constraints. The more facts a fact is contradictory with, the more likely it is a potential error. We define the contradictoriness score of a fact *t*_*i*_ as follows.

(17) $$\begin{array}{c} Contradictoriness\left(\;t_{i}\;\right)=1+\underset{j}{\sum }n_{j}\left(\;t_{i}\;\right), \end{array}$$

where *n*_*j*_(*t*_*i*_) is the number of violated ground rules of *F*_*j*_ when *t*_*i*_ appears in *F*_*j*_. The addition of 1 ensures the contradictoriness scores are greater than zero.

For example, considering a semantic constraint (rule) *F*_1_ (defined in Section 4.1), supposing there are two ground atoms *Mut*(*country*, *bird*) and *Mut*(*city*, *bird*), and three facts *Cat*(*Italy*, *country*), *Cat*(*Italy*, *city*), and *Cat*(*Italy*, *bird*), then the fact *Cat*(*Italy*, *bird*) violates two ground rules of *F*_1_. Hence, *n*_1_(*Cat*(*Italy*, *country*)) = 2.

Combining the above two factors, we use the following function to rank candidate facts.

(18) Scoreti=Uncertaintyti∗Contradictorinessti.

Based on ranking scores, we select a batch of candidate facts to conduct crowdsourcing at a time.  Algorithm 1 describes the overall procedure for refining a knowledge base. Given an extracted knowledge base and a set of semantic constraints (SCs), it first initializes confidences of candidate facts being correct with machine-based estimations and calculates scores using (15)–(18). Then, it selects *B* candidate facts ∆_*B*_ from the knowledge base (*K*) to conduct crowdsourcing where *B* is a budget allowed for improving the knowledge base.

### 4.3. Leveraging Semantic Constraints Pruning Unnecessary Questions

In this section, we discuss how to utilize semantic constraints as inference rules to reduce the crowdsourcing cost. The rank-based method discussed above simply selects top *B* candidate facts to conduct crowdsourcing at a time. However, by leveraging semantic constraints, we can infer the correctness of a candidate fact from other facts without acquiring the intelligence from the crowd. Thus, we can effectively use the budget for crowdsourcing. For example, if *child*(*x*, *y*) is correct, we do not need to crowdsource the candidate fact *parent*(*y*, *x*) since it can be inferred to be correct based on the inversion constraint (12). If *countrycapital*(*x*, *y*) is correct, we can infer any *countrycapital*(*x*, *z*) is incorrect based on the functionality constraint (7).

#### 4.3.1. Graph-Based Algorithm

To leverage semantic constraints, we model the selected candidate facts (under a given budget) for crowdsourcing as a graph based on ground inference rules and try to infer the correctness of some candidate facts using the graph model.

**Definition 2 (graph model).**

Given a set of candidate facts, we build a directed graph *G* = (*V*, *ℰ*), where each vertex in *V* is a candidate fact, *ℰ* = *ℰ*_*p*_ ∪ *ℰ*_*c*_, where *ℰ*_*p*_ represents all positive relations and *ℰ*_*c*_ represents all contradictive relations. Given two candidate facts *t*_*i*_ and *t*_*j*_, if *t*_*i*_⇒*t*_*j*_, there is a directed edge *e* ∈ *ℰ*_*p*_ from *t*_*i*_ to *t*_*j*_ to represent this positive relation; if *t*_*i*_⇒¬*t*_*j*_, there is a directed edge in *ℰ*_*c*_ from *t*_*i*_ to *t*_*j*_ to represent this contradictive relation.

**Figure 1:**
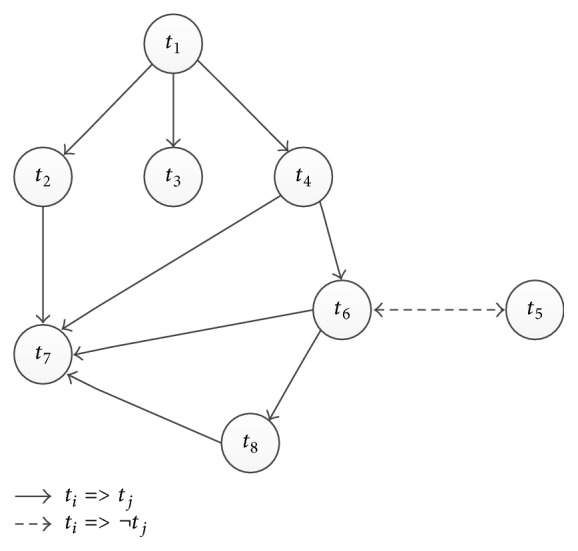
A sample of graph model.

Figure 1 shows the graph for candidate facts in Box 2. We use *G*_*p*_ = (*V*, *ℰ*_*p*_) to denote the subgraph containing only edges in *ℰ*_*p*_ and *G*_*c*_ = (*V*, *ℰ*_*c*_) to denote the subgraph containing only edges in *ℰ*_*c*_.

*Graph Coloring.* Each vertex in *G* has two possibilities: (1) the candidate fact is correct and we color it Green; (2) the candidate fact is incorrect and we color it Red. Initially, each vertex is uncolored. Our goal is to utilize the crowd to color all vertices.

A straightforward method is to take the candidate fact on each vertex as a question and ask workers to answer the question, that is, whether the candidate fact is correct. If a worker thinks that the candidate fact is correct, the worker returns Yes and No otherwise. Based on the workers' results, we get a voted answer on each vertex. If majority of workers vote Yes, we color it Green; otherwise we color it Red. Next, we interchangeably use vertex, fact, and question if the context is clear.

This method is rather expensive as there are many vertices in the graph. To address this issue, we propose an effective coloring framework to reduce the number of questions.  Algorithm 2 shows the pseudocode. It first constructs a graph based on ground inference rules (line 1-2). Then it selects an uncolored vertex *t*_*i*_ and asks workers to answer Yes or No for the vertex (line 4). If majority of workers vote Yes, we not only color *t*_*i*_ Green, but also color all of its descendants in *G*_*p*_(*desc*_*p*_(*t*_*i*_)) Green and color all of their children in *G*_c_ (*child*_*c*_(*t*_*i*_) and *child*_*c*_(*desc*_*p*_(*t*_*i*_))) Red and their ancestors in *G*_*p*_ (*ance*_*p*_(*child*_*c*_(*t*_*i*_)) and *ance*_*p*_(*child*_*c*_(*desc*_*p*_(*t*_*i*_)))) Red (line 6–8). In other words, for *t*_*i*_⇒*t*_*j*_, we can infer that *t*_*j*_ is also correct; for *t*_*i*_⇒¬*t*_*j*_, we can infer *t*_*j*_ is incorrect. If majority of workers vote No, we not only color *t*_*i*_ Red, but also color all of its ancestors in *G*_*p*_ (*ance*_*p*_(*t*_*i*_)) Red (line 10). In other words, for *t*_*j*_⇒*t*_*i*_, we infer that *t*_*j*_ is also incorrect. If all the vertices have been colored, the algorithm terminates. Otherwise, it selects an uncolored vertex and repeats the above steps (line 3–12).

Obviously, this method can reduce the crowdsourcing cost as we can avoid asking questions for many unnecessary vertices. For example, considering the constructed graph in Figure 1, a naive method is to conduct crowdsourcing for all eight facts. However, if we first conduct crowdsourcing for *t*_6_, as majority of workers vote Yes, we can color *t*_6_ and their descendants *t*_7_ and *t*_8_ Green and color *t*_5_ Red without conducting crowdsourcing for its descendants. Then if we continue to conduct crowdsourcing for *t*_4_, as majority of workers vote No, we can color *t*_4_ and its ancestor *t*_1_ Red.

An important problem in the algorithm is to select the minimum number of vertices to conduct crowdsourcing, so that all vertices in the graph are colored. We will first formulate the question selection problem and then propose a path-based algorithm and a topological-sorting-based algorithm that select multiple vertices in each iteration to solve the problem.

#### 4.3.2. Optimal Vertex Selection

As we know, we have the basic coloring strategy: if a vertex is Green, then all of its descendants in *G*_*p*_ are Green but all of its children in *G*_*c*_ and their ancestors in *G*_*p*_ are Red; if a vertex is Red, then all of its ancestors in *G*_*p*_ are Red. We will discuss how to support the case that the two conditions do not hold in Section 4.4.

**Definition 3 (optimal graph coloring).**

Given a graph, the optimal graph coloring problem aims to select the minimum number of vertices as questions to color all the vertices using the coloring strategy.

**Figure 2:**
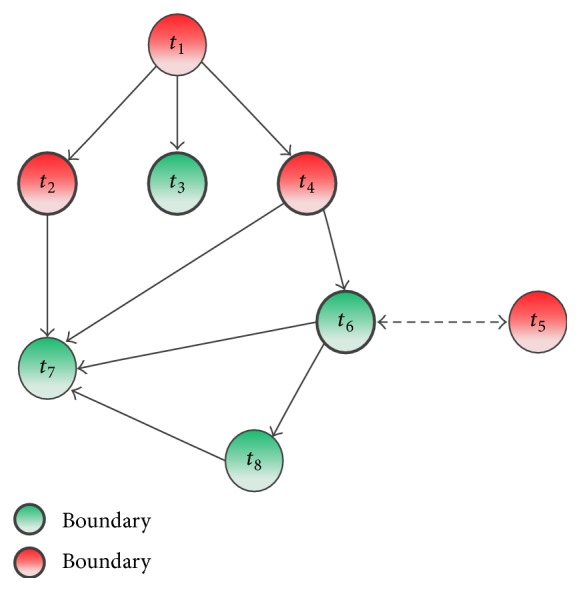
A sample of boundary vertex.

For example, in Figure 2, if we sequentially select vertices *t*_1_, *t*_2_, *t*_3_, *t*_4_, *t*_5_, *t*_7_, and *t*_6_, we should ask seven questions. The optimal crowdsourced vertices are *t*_2_, *t*_3_, *t*_4_, and *t*_6_ (highlighted by bold cycles), because the colors of these vertices cannot be inferred based on the colors of other vertices. Next we will study how to identify the optimal vertices. Before that, we introduce a concept for making our explanation easily.

**Definition 4 (boundary vertex).**

A vertex is a boundary vertex if its color cannot be inferred based on other vertices' colors. There are four cases in *G*_*p*_: (1) all of its parents have different colors with the vertex; (2) all of its children have different colors with the vertex; (3) it has no parent and its color is Green; or (4) it has no children and its color is Red. In addition, there are two cases in *G*_*c*_: (1) all of its parents are Red; (2) it has no parents (in-edge).

For example, *t*_6_ is a boundary vertex in *G*_*p*_ as its parent *t*_4_ has a different color. *t*_8_ is not a boundary vertex as its parent *t*_6_ has the same color and *t*_8_'s color can be inferred based on *t*_6_'s color. *t*_6_ is also a boundary vertex in *G*_*c*_ as its parent *t*_5_ is Red.

Here we use *V*_*B*_ to denote all the boundary vertices in the graph and *V*_*B*_(*G*_*p*_) and *V*_*B*_(*G*_*c*_) to denote the boundary vertices in *G*_*p*_ and *G*_*c*_, respectively. There are overlaps between *V*_*B*_(*G*_*p*_) and *V*_*B*_(*G*_*c*_), so *V*_*B*_ = *V*_*B*_(*G*_*p*_)∩*V*_*B*_(*G*_*c*_). Ideally, all vertices in *V*_*B*_ should be checked, because their colors cannot be inferred. Thus, the number of vertices checked using any algorithm should not be smaller than the number of boundary vertices in *V*_*B*_. However, since we do not know the ground truths of all vertices in the graph, we cannot identify the boundary vertices in advance. To address this problem, we propose effective algorithms to identify the boundary vertices in *G*_*p*_ with a theoretical guarantee and use a greedy algorithm to identify the boundary vertices in *G*_*c*_, respectively. Meanwhile, we note that there are more boundary vertices in *G*_*c*_ because of the limited influence of a vertex in *G*_*c*_ than those in *G*_*p*_. Hence, we consider firstly the boundary vertices in *G*_*p*_.

*Optimal Vertex Selection in G*_*p*_. Given a path in *G*_*p*_, we can use a binary search method to select the boundary vertices. We initially crowdsource the mid-vertex on the path. Based on the result of the mid-vertex, we determine the next step. There are two situations: (1) If the mid-vertex is colored Green, its descendants' colors can be inferred but its ancestors' colors can not be inferred. Thus, we can crowdsource the next mid-vertex between the current vertex and the source vertex of the path. (2) If the mid-vertex is colored Red, its ancestors' colors can be inferred but its descendants' colors can not be inferred. Therefore, we can crowdsource the next mid-vertex between the current vertex and the destination vertex of the path. Iteratively, we can find all the boundary vertices. For the path *P* with |*P*| vertices, the number of crowdsourcing vertices is *O*(log⁡|*P*|).

*Optimal Vertex Selection in G*_*c*_. We greedily select the vertices with no in-edge in *G*_*c*_ and the vertices with the largest confidence value in each contradictive group (i.e., connected subgraph), since only Green vertices can be used to infer colors of its children.

#### 4.3.3. Path-Based Algorithm

We can divide the graph *G*_*p*_ into a set of disjoint paths (i.e., any two paths have no common vertices). Then we can use the binary search method described above to determine the vertices for crowdsourcing. As the maximum length of a path is |*V*|, the number of crowdsourcing vertices is *O*(*β*log⁡|*V*|), where *β* is the number of disjoint paths. If *β* = 1, we need to crowdsource log⁡|*V*| vertices.

*Finding β Disjoint Paths*. In order to find the disjoint paths, we transform the graph *G*_*p*_ into a bipartite graph *G*_*p*_^*b*^ = ((*V*_1_^*b*^, *V*_2_^*b*^), *ℰ*_*p*_^*b*^), where *V*_1_^*b*^ = *V*_2_^*b*^ = *V* and there is an edge between *v*_1_ ∈ *V*_1_^*b*^ and *v*_2_ ∈ *V*_2_^*b*^ if there is an edge (*v*_1_, *v*_2_) ∈ *ℰ*_*p*_. We find maximal matching in *G*_*p*_^*b*^, which is a maximal set of edges in *G*_*p*_^*b*^ where any two edges do not share a common vertex in *V*_1_^*b*^ and *V*_2_^*b*^. That is, for any two edges (*v*, *v*′), (*u*, *u*′) in the matching, *v* ≠ *u* and *v*′ ≠ *u*′. Obviously, any two edges in the matching sharing the same vertex in *V* must be on the same path. Based on this idea, we utilize the maximal matching to find the *β* disjoint paths as follows.

Let *M* denote the maximal matchings, *Y*_1_ denote the set of the first vertices in *M* and *Y*_2_ denote the set of the second vertices in *M*. Then *V*_2_^*b*^ − *Y*_2_ is the set of vertices that have no in-edges, and we can take them as the first vertices of paths. For each such a vertex *v*, if it has an edge (*v*, *v*′), we take *v*′ as the second vertex in a path. Then we check whether *v*′ has an edge (*v*′, *v*′′). Iteratively, we can find the path starting at *v*. The paths computed using maximal matching satisfy disjoint, complete, and minimal paths [30].

Then we propose a serial path-based vertex-selection algorithm. The pseudocode is shown in Algorithm 3. It computes disjoint paths in *G*_*p*_ using maximal matching and selects the optimal vertex of the longest path to conduct crowdsourcing. When there is no path with length greater than 1, it selects an optimal vertex according to *G*_*c*_.

The serial path-based vertex-selection algorithm can only publish a single fact to a crowdsourcing platform at a time, which is unable to crowdsource candidate facts simultaneously and results in long latency. To overcome this drawback, we extend the path-based algorithm to support parallel settings, which select multiple vertices and publish the corresponding candidate facts simultaneously to the crowdsourcing platform in each iteration. The pseudo code is shown in Algorithm 4. We first identify the *β* disjoint paths and use the optimal vertex selection strategy discussed in Section 4.3.2 to select one vertex from each path to conduct crowdsourcing in parallel. When there is no path with length greater than 1, we select the optimal vertices according to *G*_*c*_. Based on the answers of these vertices, we color the graph. Next we remove the colored vertices and repeat the above step until all the vertices are colored.

However, the parallel algorithm may generate conflicts. For example, if *t*_*i*_ is colored Green and *t*_*j*_ is colored Red, then there is a conflict on *t* where *t*_*i*_⇒*t* and *t*⇒*t*_*j*_, because *t* is inferred as Green based on *t*_*i*_ but is inferred as Red based on *t*_*j*_. To address this confliction, we can use majority voting to vote *t*'s color and randomly choose one if a tie occurred.

#### 4.3.4. Topological-Sorting-Based Algorithm

Note that the maximal matching can be computed in *O*(*β*|*V*|^2^) [30], which is too slow in practice when used for a large knowledge base. To address this issue, we perform a topological sorting on the vertices. We first identify the set of vertices with zero in-degree, denoted by *L*_1_. Then we delete them from the graph and find another set of vertices whose in-degrees are zero, denoted by *L*_2_. We repeat this step until all vertices are deleted. Suppose there are |*L*| sets, *L*_1_, *L*_2_,…, *L*_|*L*|_. Obviously vertices in each *L*_*i*_ have no in-edges (as their in-degrees are 0). Therefore, each *L*_*i*_ can be considered as an independent set.

We design a topological-sorting-based algorithm to improve the time efficiency of the maximal matching. It first computes topological-sorted sets *L*_1_, *L*_2_,…, *L*_|*L*|_ in *G*_*p*_. And then it crowdsources vertices in the middle set *L*_(|*L*| + 1)/2_ in parallel. When |*L*| ≤ 1, it selects optimal vertices according to *G*_*c*_ to conduct crowdsourcing. Based on the results of these vertices, it colors the graph and removes the colored vertices and *L*_(|*L*| + 1)/2_ from the set *L*. It repeats the above step and iteratively colors all vertices. The pseudo code of the topological-sorting-based algorithm is shown in Algorithm 5.

### 4.4. Tolerating Errors

There are two types of possible errors in our graph-based framework. The first type is caused by workers' errors and the second type is propagated through inference rules. For example, suppose a candidate fact *t*_*i*_ is actually incorrect. However, the workers wrongly label it as correct. This error is caused by workers' errors. Consider a contradictive fact *t*_*j*_ of *t*_*i*_, whose labels are correct. Our graph-based algorithms could wrongly label it as incorrect using inference rules. This error is propagated through inference rules. We will discuss how to address these errors in our framework as follows.

*Confidence of Workers' Answer.* To tolerate workers' errors, we assign each candidate fact to multiple workers and aggregate their answers. There are many methods to compute the confidence of workers' answers. We use majority voting as an example and any other techniques can be integrated into our framework. Suppose each candidate fact is assigned to *z* workers and *y* > *z*/2 workers vote a consensus answer (e.g., Yes) and *z* − *y* workers vote the other answer (e.g., No). The confidence of the voted answer is *c* = *y*/*z*.

*Error-Tolerant Coloring.* For each crowdsourced fact, if the confidence of workers on this fact is high, for example, greater than 0.8, we use inference rules to label related candidate facts; otherwise, we label it uncertain (color the vertex in graph as Blue) and do not use it to infer the labels of other candidate facts. For the Green and the Red vertices, we take them as ground truths as their answers have large confidences. Then we utilize them to color Blue (uncertain) vertices. Specifically, we use facts in Green and Red vertices to learn a logistic regression model on the machine confidence scores (provided by the information extraction system) and predict the labels of facts in Blue vertices.

The pseudo code of our error-tolerant coloring algorithm in shown in Algorithm 6. It uses the coloring strategy only for the vertices with high-confidence answers (line 5) and utilizes the logistic regression model to color the vertices with low-confidence answers (lines 11-12).

## 5. Experiments

In this section, we evaluate our methods and report experimental results.

### 5.1. Experimental Setup

*Datasets.* NELL [4] generates a knowledge base iteratively. In each iteration, NELL uses facts learned from the previous iteration and a corpus of web pages to generate a new set of candidate facts. NELL selectively promotes those candidates that have a high confidence from the extractors and obey ontological constraints with the existing knowledge base to build a high-precision knowledge base. We use extractions of the 165*th* iteration of NELL released by [7] to evaluate our method, containing over 1 M extractions, with a manual labelled test set consisting of 4546 instances and a training set consisting of 9866 instances. There are 70 K ontological relations in total.  Table 1 shows the statistics of the data set. The training set can be used to calibrate the confidence scores from the original system. When training data is not available, we can adopt the confidence provided by the information extraction system as the probability.

We calculate contradictoriness scores among all candidate facts and select candidate facts from the test set for crowdsourcing. We use a threshold 0.5 for the confidence score. For crowdsourced data, a fact is treated as correct only when more than half of crowd answers are “Yes.” We compare our methods with other popular methods in terms of the quality, the number of questions, and the number of iterations. To evaluate the quality, we use three metrics, that is, precision, recall, and* F1*. Suppose the set of correct facts is *S*_*T*_, and the set of facts that an algorithm reports as correct is *S*_*P*_. Then the precision is *p* = |*S*_*T*_∩*S*_*P*_|/|*S*_*P*_|, the recall is *r* = |*S*_*T*_∩*S*_*P*_|/|*S*_*T*_|, and the* F*-measure is *F*1 = 2*pr*/(*p* + *r*).

*Crowdsourcing on MTurk.* We use MTurk for crowdsourcing. We post all candidate facts in the test set to MTurk and record the crowd's answers in a local file *F*. During our experiments, when a method requests to crowdsource candidate facts, we retrieve answers from *F* instead of posting facts to MTurk. This ensures that all methods utilize the same set of crowdsourced results, for the fairness of comparisons. We take four microtasks as one HIT, where each microtask contains a candidate fact. To assist workers to understand the fact, we provide a description of each category or relation and use a human format for each entity (see Box 1 as an example). We pay $0.02 each time a worker completes an HIT and $0.01 to MTurk for publishing each HIT. We assign each HIT to five workers. We require that each worker has an approval rate greater than 95%. This setting intends to ensure that all workers provide reasonably accurate answers to the HITs.

### 5.2. Experimental Results

We first compare our method with state-of-the-art methods for knowledge refining. Then we evaluate our rank function, question selection strategies, and error-tolerant techniques, respectively.

#### 5.2.1. Evaluation of Our Methods

In order to evaluate the effectiveness of our proposed techniques, we compare our methods Rank, Graph, and Graph+ (graph-based method with error-tolerant techniques) with two recent methods for cleaning automatically extracted knowledge bases, that is, MLN [8] and PSL [7], using previously reported results on the same evaluation set. We also compare with the default strategy used by the NELL [4] project to choose candidate facts to include in the knowledge base.

*MLN [8]*. This method defines a Markov logic network (MLN) to perform jointly probabilistic inference over candidate facts. We compare our method against the best-performing MLN model from [8], which expresses ontological constraints, and candidate and promoted facts trough logical rules. The MLN method reports an output with a 0.5 marginal probability cutoff, which maximizes the* F1* score.

*PSL [7]*. This method uses probabilistic soft logic (PSL) to jointly reason candidate facts and identify coreferent entities, which can perform inference more efficiently. The PSL method reports results using a soft-truth threshold 0.55 to maximize* F1*.

*NELL [4]*. We also compare the default strategy used by the NELL project to choose candidate facts to include in the knowledge base. We take the promoted facts as its result.

**Figure 3:**
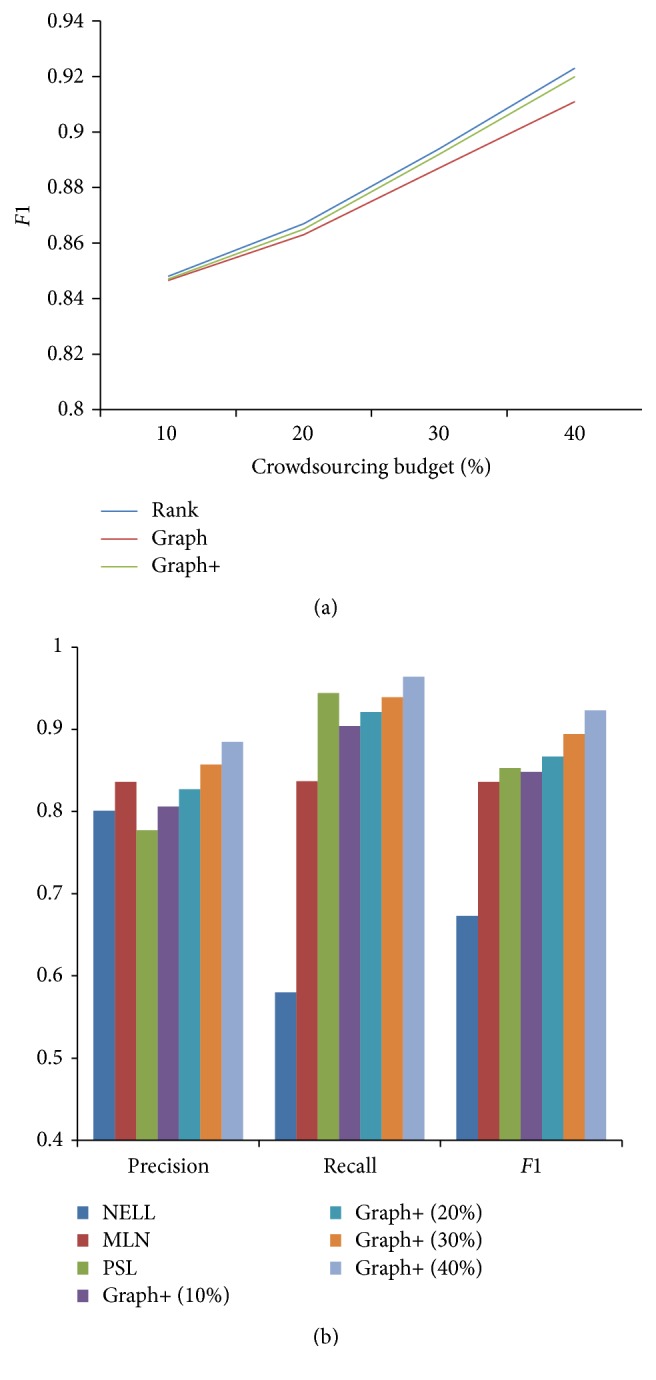
Evaluation of proposed methods. (a) Quality comparison of proposed methods for different crowdsourcing budgets. (b) Quality comparison with the state-of-the-art methods.

Given a budget *B* (e.g., 40% of candidate facts), our Rank method selects top *B* candidate facts to conduct crowdsourcing at a time. For the Graph and Graph+ methods, we construct a graph with the top *B* candidate facts and use the topological-sorting algorithm to select questions for crowdsourcing.  Figure 3(a) shows a comparison of the overall performance of our Rank, Graph, and Graph+ methods. We report the results of our methods under different budgets. From Figure 3(a), we can see that, given a larger crowdsourcing budget, our method can obtain a higher performance. Graph+ and Rank achieve a similar quality. However, Graph+ asks fewer questions than Rank, as shown in Section 5.2.3. Graph+ outperforms Graph, because Graph+ can tolerate workers' errors by not coloring unconfident vertices and thus avoids enlarging the errors by a wrong colored vertex.  Figure 3(b) shows a comparison of the overall performance with the state-of-the-art methods. From Figure 3(b), we can see that MLN and PSL perform well in precision or recall, respectively. Our method improves both precision and recall. Overall, our method improves significantly on* F1*. With a reasonable budget (above 20% test instances), our method outperforms both MLN and PSL methods in terms of* F1*.

#### 5.2.2. Evaluation on Rank Function

In this experiment, we evaluate our ranking function, which is a key for selecting crowdsourcing candidate facts in the rank-based method. This function, denoted as U*∗*C, quantifies the usefulness of a candidate fact by considering both its uncertainty and its contradictoriness with other facts. We compare U*∗*C against following baselines. (1) Method* Uncertainty* considers only uncertainty scores. (2) Method *Contradictoriness* considers only contradictoriness scores. (3) Method* Random* selects candidate facts for crowdsourcing randomly.

**Figure 4:**
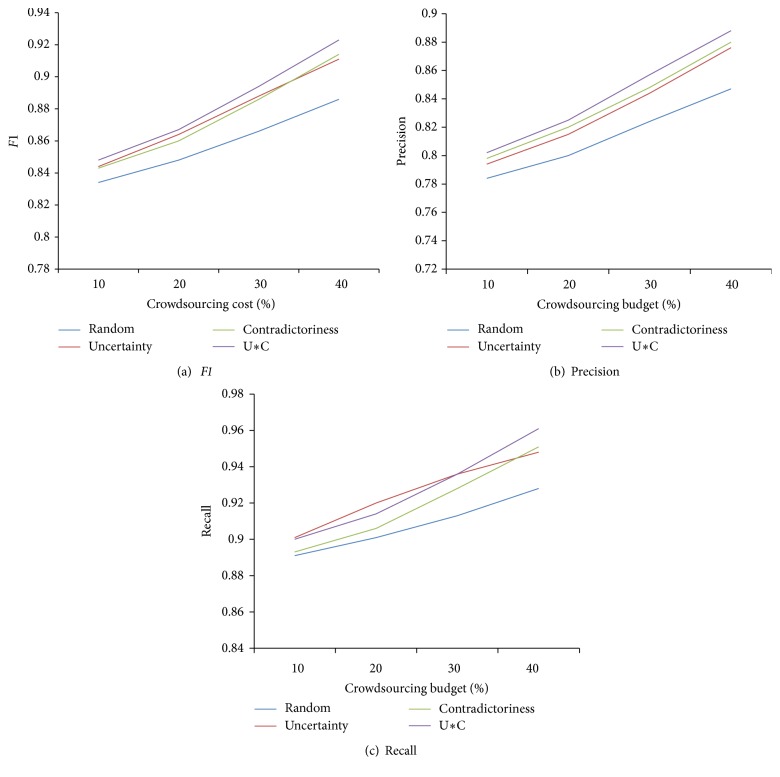
Evaluation of different ranking functions.

Figure 4 shows the results of* F1* using different ranking functions. The U*∗*C method achieves the highest* F1*. The Uncertainty method achieves the highest recall at the beginning but the speed of improvement slowing down with the increment of the budget. This is because there are many false positives and negatives among candidate facts with confidences around the threshold, which have higher uncertainty scores. The error rate drops quickly when the difference between confidence and threshold increases, while considering contradictoriness can still help detect potential erroneous facts effectively. The *Contradictoriness* method achieves better precision than *Uncertainty*. *Random* consistently performs the worst.

#### 5.2.3. Evaluation on Question Selection

**Figure 5:**
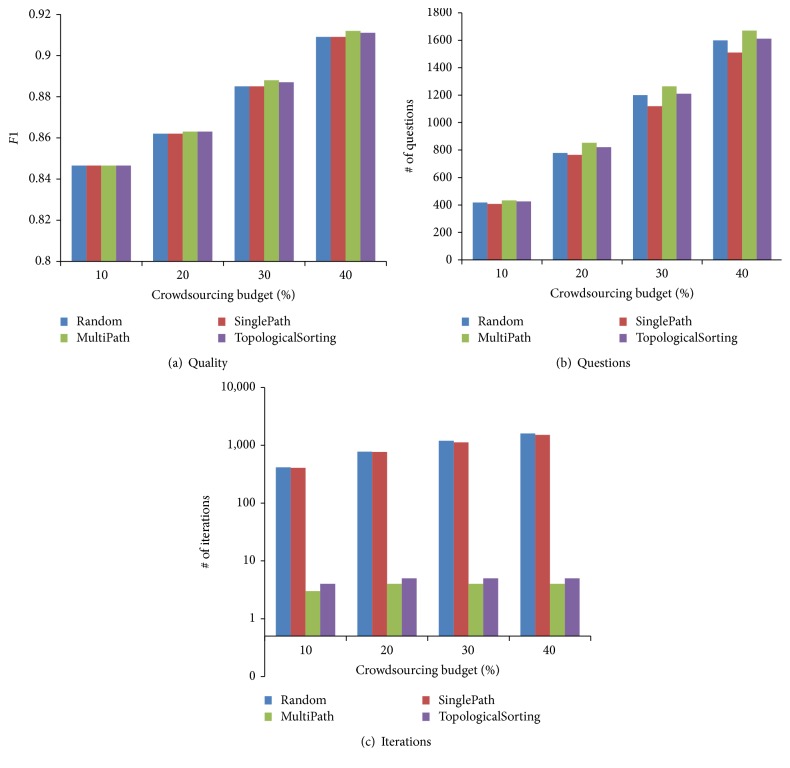
Evaluation of question selection strategies on the test dataset.

From Section 5.2.1, we can see that the Graph+ method has a similar quality with the Rank method. In this section, we focus on the efficiency of question selection algorithms in terms of the number of questions and the number of iterations. We evaluate the path-based and topological-sorting-based question selection algorithms proposed in Sections 4.3.3 and 4.3.4. We compare four algorithms: (1) Random: which randomly selects a vertex in each iteration. (2) SinglePath: which selects a vertex from the longest path in each iteration. (3) Multipath: which selects multiple vertices from multiple disjoint paths in each iteration. (4) TopologicalSorting: which selects multiple independent vertices based on topological sorting in each iteration. We compare them in terms of the quality, the number of questions, and the number of iterations, shown in Figure 5.

From Figure 5(a), we can see that the four methods achieve the similar quality, because different question orders do not affect the quality based on inference rules. From Figure 5(b), we can see that the two parallel algorithms Multipath and TopologicalSorting crowdsource a few more questions than SinglePath. This is because Multipath may crowdsource vertices with ancestor-descendant relationships and TopologicalSorting may crowdsource vertices with the same descendants which can be avoided by our serial algorithm SinglePath based on the inference rules. TopologicalSorting outperforms Multipath because TopologicalSorting crowdsources independent questions in each iteration while Multipath may crowdsource dependent questions. SinglePath outperforms Random and reduces the number of questions. This is because SinglePath can effectively identify the boundary vertex using the optimal vertex search strategy. From Figure 5(c), the two parallel algorithms Multipath and TopologicalSorting significantly outperform SinglePath and Random as they crowdsource questions in parallel.

**Figure 6:**
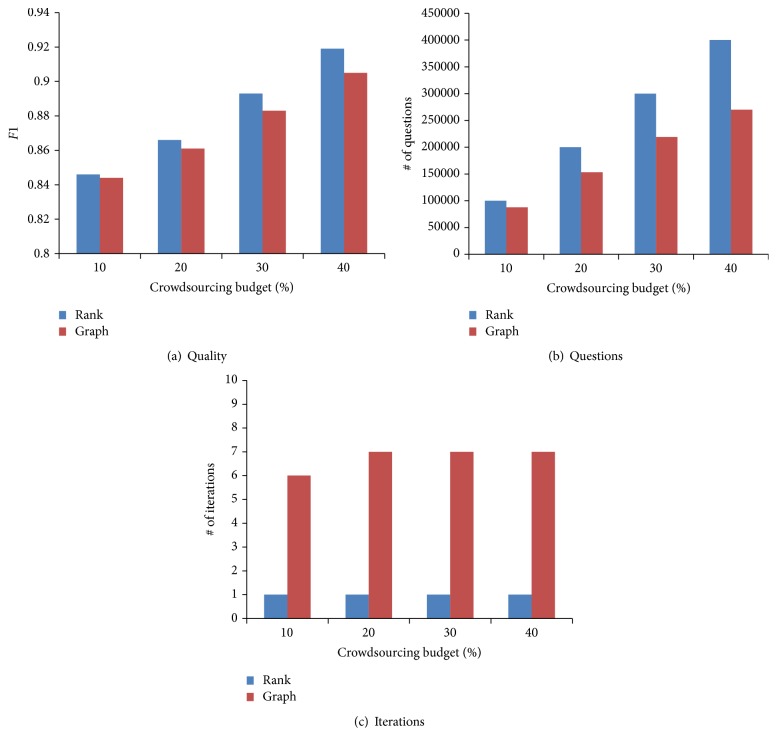
Evaluation of question selection strategies on the complete dataset.

To evaluate our graph-based method (Graph) on reducing the number of questions, we conduct additional simulation experiments on the complete dataset, using NELL beliefs as ground truths and simulating workers with accuracy of 90%. Our experimental results are shown in Figure 6. Figure 6 shows that our graph-based method crowdsources fewer questions than our rank-based method (Rank). It saves even more than 30%, comparing with the rank-based method. This is because we can utilize the inference rules to prune many candidate facts that do not need to be crowdsourced. The rank-based method achieves a higher quality at the expense of crowdsourcing many more questions. Besides, the graph-based method only involves a few iterations, because it can crowdsource many questions in parallel.

#### 5.2.4. Evaluation on the Error-Tolerant Solution

**Figure 7:**
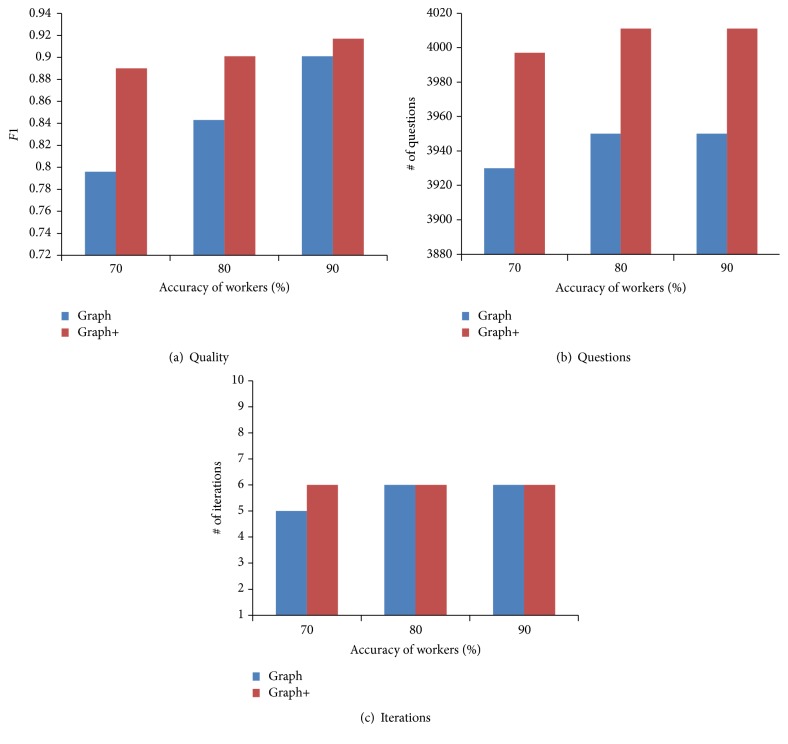
Evaluation of our error-tolerant technique.

In this section, we evaluate the effectiveness of our error-tolerant solution (proposed in Section 4.4) by comparing two algorithms: (1) Graph: which does not consider errors; (2) Graph+: which extends Graph to tolerate errors. We use simulated workers and conduct evaluation under different accuracy levels of crowdsourcing workers (i.e., 70%, 80%, and 90%) on test dataset. We compare Graph+ with Graph in terms of quality, the number of questions, and the number of iterations. Our experimental results are shown in Figure 7.

From Figure 7, we can see that Graph+ achieves a higher quality than Graph, because it can tolerate the errors introduced by crowdsourcing workers and avoid error propagation along the inference rules. Graph+ significantly outperforms Graph for low-quality workers. With the increment of the accuracy level of workers, the improvement decreases. On the other hand, Graph+ crowdsources a little more questions than Graph. This is because Graph+ does not utilize the inference rules for some facts, so that it reduces the number of inferred facts. From Figure 7(c), we can see that the two methods have the same number of iterations. This is expected, since the only difference between Graph+ and Graph is that Graph+ does not infer the answers for some unconfident facts. The accuracy level of crowdsourcing workers has little impact on the number of questions and the number of iterations for both methods, because the number of questions and the number of iterations are determined by the graph structure. Therefore, we can use the error-tolerant technique to improve the quality of the knowledge base.

## 6. Conclusions

We proposed a cost-effective method for cleaning automatically extracted knowledge bases using crowdsourcing. Our method uses a ranking score to select the most beneficial candidate facts for crowdsourcing in terms of improving the quality of knowledge bases. We constructed a graph based on the semantic constraints and utilized the graph to crowdsource questions and infer answers. We evaluated the effectiveness of our methods on real-world web extractions from NELL. Our experimental results showed that our method outperforms both MLN-based and PSL-based methods in terms of* F1* under a reasonable crowdsourcing cost.

## Figures and Tables

**Box 1:**
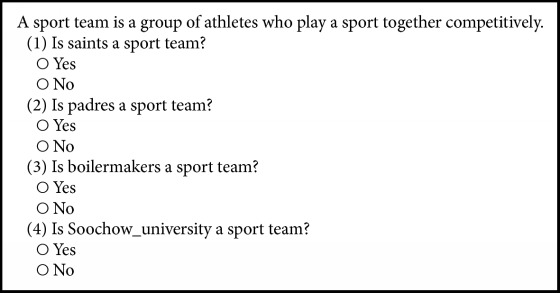
An example of HITs.

**Box 2:**
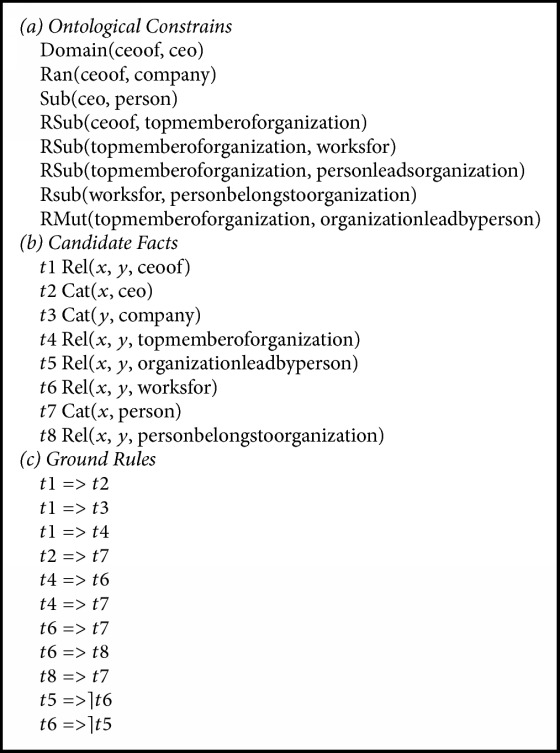
Sample semantic constraints in NELL.

**Algorithm 1:**
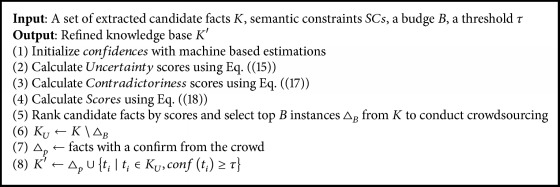
Rank-based knowledge refining.

**Algorithm 2:**
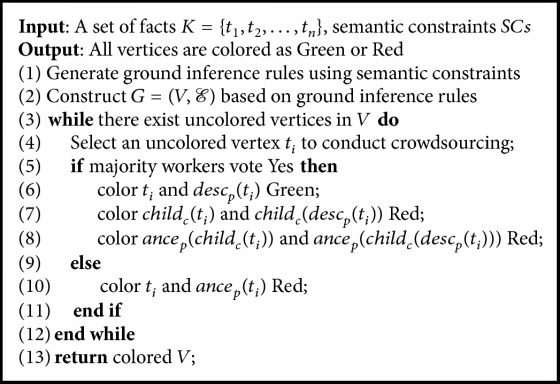
Graph coloring.

**Algorithm 3:**
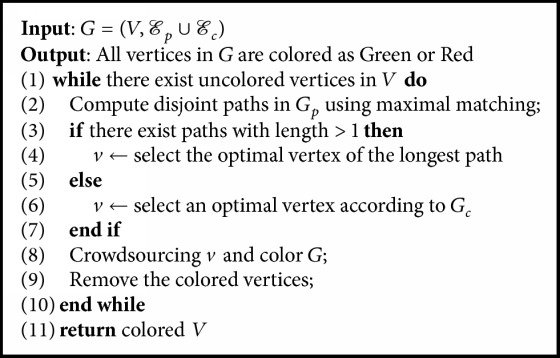
Question selection: SinglePath.

**Algorithm 4:**
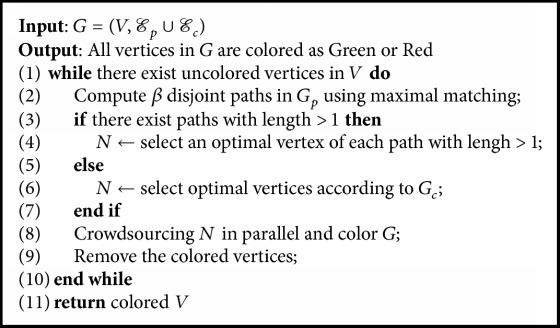
Question selection: Multi-Path.

**Algorithm 5:**
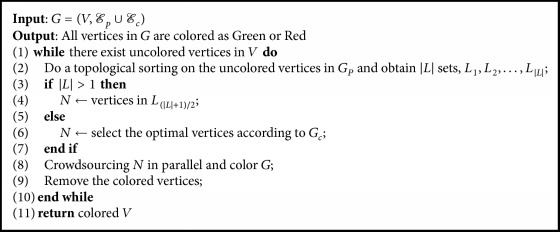
Question selection: TopologicalSorting.

**Algorithm 6:**
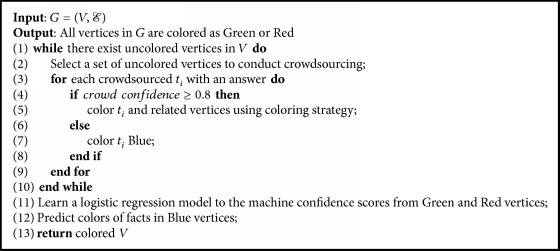
Error-tolerant graph coloring.

**Table 1:** Statistic characters of dataset.

Dataset	Category	Relation	Total
Candidate	836K	182K	1.02M
Promotion	354K	64K	418K
Ontological Relation	18K	52K	70K
Test	2002	2546	4546
Training	4777	5089	9866
